# Effect of withholding early parenteral nutrition in PICU on ketogenesis as potential mediator of its outcome benefit

**DOI:** 10.1186/s13054-020-03256-z

**Published:** 2020-08-31

**Authors:** Astrid De Bruyn, Jan Gunst, Chloë Goossens, Sarah Vander Perre, Gonzalo G. Guerra, Sascha Verbruggen, Koen Joosten, Lies Langouche, Greet Van den Berghe

**Affiliations:** 1 Clinical Division and Laboratory of Intensive Care Medicine, Department of Cellular and Molecular Medicine, KU Leuven, 3000 Leuven, Belgium; 2 Department of Paediatrics, Intensive Care Unit, University of Alberta, Stollery Children’s Hospital, Edmonton, AB Canada; 3 Intensive Care Unit, Department of Paediatrics and Paediatric Surgery, Erasmus Medical Centre-Sophia Children’s Hospital, Rotterdam, Netherlands

**Keywords:** Parenteral nutrition, Intensive care, Ketogenesis, Ketone body, Recovery

## Abstract

**Background:**

In critically ill children, omitting early use of parenteral nutrition (late-PN versus early-PN) reduced infections, accelerated weaning from mechanical ventilation, and shortened PICU stay. We hypothesized that fasting-induced ketogenesis mediates these benefits.

**Methods:**

In a secondary analysis of the PEPaNIC RCT (*N* = 1440), the impact of late-PN versus early-PN on plasma 3-hydroxybutyrate (3HB), and on blood glucose, plasma insulin, and glucagon as key ketogenesis regulators, was determined for 96 matched patients staying ≥ 5 days in PICU, and the day of maximal 3HB-effect, if any, was identified. Subsequently, in the total study population, plasma 3HB and late-PN-affected ketogenesis regulators were measured on that average day of maximal 3HB effect. Multivariable Cox proportional hazard and logistic regression analyses were performed adjusting for randomization and baseline risk factors. Whether any potential mediator role for 3HB was direct or indirect was assessed by further adjusting for ketogenesis regulators.

**Results:**

In the matched cohort (*n* = 96), late-PN versus early-PN increased plasma 3HB throughout PICU days 1–5 (*P* < 0.0001), maximally on PICU day 2. Also, blood glucose (*P* < 0.001) and plasma insulin (*P* < 0.0001), but not glucagon, were affected. In the total cohort (*n* = 1142 with available plasma), late-PN increased plasma 3HB on PICU day 2 (day 1 for shorter stayers) from (median [IQR]) 0.04 [0.04–0.04] mmol/L to 0.75 [0.04–2.03] mmol/L (*P* < 0.0001). The 3HB effect of late-PN statistically explained its impact on weaning from mechanical ventilation (*P* = 0.0002) and on time to live PICU discharge (*P* = 0.004). Further adjustment for regulators of ketogenesis did not alter these findings.

**Conclusion:**

Withholding early-PN in critically ill children significantly increased plasma 3HB, a direct effect that statistically mediated an important part of its outcome benefit.

## Introduction

Critically ill patients treated in the pediatric intensive care unit (PICU) often develop a pronounced macronutrient deficit because of the inability to feed orally and because nutrition administered via nasogastric tubes is poorly tolerated. The degree of the accumulated macronutrient deficit has been associated with poor outcome and delayed recovery [1, 2]. However, this association is confounded by illness severity, given that the sicker patients are the ones to poorly tolerate enteral feeding. Furthermore, recent randomized controlled trials (RCTs) with variable design have not shown unequivocal benefit by early supplementation of insufficient or failing enteral nutrition, whereby some RCTs even indicated potential harm [3–7]. Indeed, the Early versus Late Parenteral Nutrition in Critically Ill Adults (EPaNIC) and Paediatric Early versus Late Parenteral Nutrition In Critical Illness (PEPaNIC) RCTs have shown that withholding parenteral nutrition until beyond the first week in intensive care (late-PN) was clinically superior to early parenteral nutrition supplementing insufficient enteral nutrition (early-PN) [4, 5]. As compared with early-PN, late-PN shortened dependency on intensive medical care in both RCTs, with a shorter duration of mechanical ventilatory support, fewer newly acquired infections, and a shorter intensive care and hospital stay [4, 5]. In critically ill adults, late-PN also was found to lower the incidence of weakness [8]. Nevertheless, the optimal timing of initiating PN remains unclear.

One potential mediator of these clinical benefits brought about by accepting a macronutrient deficit early during critical illness is induction of a ketogenic fasting response. In healthy individuals, a sustained macronutrient deficit induces a fasting response with activated lipolysis and increased ketogenesis, together resulting in increased formation of the ketone bodies 3-hydroxybutyrate (3HB) and acetoacetate [9]. Apart from being a vital, energy-efficient alternative fuel for the brain, heart, and skeletal muscle in times of fasting, ketone bodies also play an important signaling role. Ketone bodies enhance autophagy-driven cellular housekeeping and activate muscle regeneration [10, 11], pathways that have been shown to be hampered or insufficiently activated during critical illness [12, 13]. In addition, ketone bodies have anti-inflammatory properties [14]. Ketone bodies or ketogenic diets have shown to increase endurance in healthy athletes [15] and to induce beneficial effects in animal models of brain injury [16]. Moreover, in a mouse model of sepsis, a ketogenic PN formula and supplementation of PN with 3HB have shown to protect against the development of muscle weakness [17].

It remains unknown, however, whether critically ill patients can increase ketogenesis in response to the illness-associated macronutrient deficit, and whether such ketogenic response—if present—is beneficial for recovery. In healthy subjects, fasting-induced ketogenesis is mediated by low circulating levels of insulin and glucose as well as high levels of glucagon, cortisol, and catecholamines [18–20]. In critical illness, however, although plasma glucagon, cortisol, and catecholamines are elevated, concomitant hyperinsulinemia and hyperglycemia may suppress ketogenesis [20, 21]. In line with this, available data, though scarce, suggested suppressed ketogenesis during critical illness [22–25]. Withholding early-PN has shown to reduce the degree of hyperglycemia, to lower the insulin requirements to prevent hyperglycemia [4, 5], and to lower the insulin/glucagon ratio [26].

We hypothesized that withholding early-PN enhances ketogenesis during critical illness and that such fasting-induced ketogenesis could be a mediator of the previously demonstrated recovery-enhancing effect of omitting PN early during critical illness [5]. We tested these hypotheses in a secondary analysis of the PEPaNIC RCT.

## Methods

### Patients and study design

This is a secondary analysis of the multicenter (Leuven, BE, Rotterdam, NL, Edmonton, CA) PEPaNIC randomized controlled trial (ClinicalTrials.gov NCT01536275, *n* = 1440). Written informed consent was obtained from the parents or legal guardians. The institutional or national ethical review boards of the participating centers approved the study protocol which was performed in accordance with the 1964 Declaration of Helsinki and later amendments. The detailed study protocol and primary results of the PEPaNIC study have been published [5, 27]. The study investigated the effect of omitting the use of PN to beyond the first week in the PICU (late-PN) as compared with the use of PN to complete insufficient or failing enteral nutrition from PICU admission onwards (early-PN). In both randomization groups, enteral nutrition was initiated as soon as possible, and gradually advanced up to target as tolerated. Beyond the first week, both groups received supplemental PN as long as patients were not fully enterally fed. To match the fluid intake of patients in the early-PN group, patients in the late-PN group received an isovolemic intravenous mixture of dextrose 5% and normal saline. To prevent refeeding syndrome, patients in both groups received intravenous trace elements, minerals, and vitamins, started on day 2 and continued until full enteral nutrition. Hyperglycemia was prevented with the use of intravenous insulin infusion, with center-specific target ranges [5, 27].

**Fig. 1:**
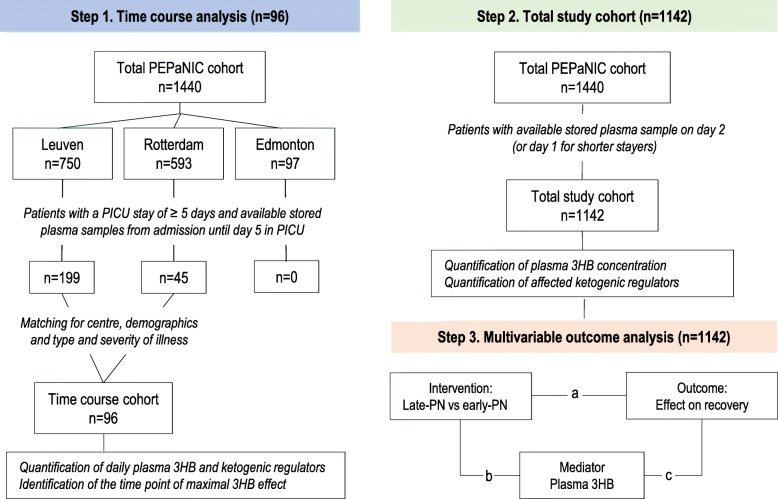
Rationale and stepwise design of the study. In step 1, a time course analysis to investigate any impact of late-PN versus early-PN on daily plasma concentrations of 3HB. Therefore, all patients with a PICU stay of at least 5 days and plasma samples available from admission until day 5 in the PICU were selected and matched for center, demographics, and type and severity of illness. In the resulting matched cohort of 96 children, daily plasma 3HB, insulin, glucagon, and glucose concentrations were determined and the time point of “maximal 3HB effect” was identified. In step 2, in the total study population, the concentration of plasma 3HB and in step 1 affected ketogenic regulators were determined on the average time point of “maximal 3HB effect” identified in step 1. For patients with a shorter PICU stay, the sample on the last day in PICU was used as surrogate (1142 patients with available plasma, of which 580 randomized to late-PN and 562 randomized to early-PN). In step 3, a multivariable statistical analysis was performed to assess whether any impact of late-PN as compared with early-PN on 3HB could explain its beneficial effects on outcome. “a” represents the effect of the intervention (late-PN versus early-PN) on the outcome of interest (time to live weaning from mechanical ventilatory support, time to live PICU discharge, and acquisition of new infection), “b” represents the effect of the intervention (late-PN versus early-PN) on the hypothesized mediator—plasma 3HB concentration, and “c” represents the association of the potential mediator with the outcome of interest (time to live weaning from mechanical ventilatory support, time to live PICU discharge, and acquisition of new infection)

First, to evaluate whether and in which timeframe late-PN versus early-PN would affect ketogenesis, a time course analysis was planned in a matched subset of patients (Fig. 1). All patients who stayed in the PICU for at least 5 days and for whom daily stored plasma samples were available from admission until day 5 in PICU were selected in the Leuven, Rotterdam, and Edmonton cohorts. For Edmonton, this resulted in 0 children; for Rotterdam, this selection resulted in a cohort of 45 children; for Leuven, this selection resulted in a cohort of 199 children. To avoid confounding of the center-specific insulin treatment strategies which could have affected plasma 3HB concentrations differently, the Leuven cohort was further reduced to a similar size as the Rotterdam cohort (*n* = 51) by propensity score matching. The resulting total cohort consisted of 96 patients (47 early-PN and 49 late-PN patients) matched for demographics (age, gender, weight) and baseline type and severity of illness (nutritional risk level according to STRONGkids category, probability of death score evaluated by PIM2 score, emergency versus planned admission, diagnostic group, need of hemodynamic assist device on admission, presence of infection on admission) (Table 1). In this set, the impact of late-PN versus early-PN on daily plasma concentrations of 3HB as well as on blood glucose, plasma insulin, and glucagon concentrations, potential regulators of ketogenesis, was determined. The time point of the maximal 3HB effect, if any, was then identified. Second (Fig. 1), for all patients in the PEPaNIC trial who had a plasma sample available for that average day of maximal effect (or last day in PICU for patients who were discharged from or died in the PICU before that day), plasma 3HB concentrations and also blood glucose, plasma insulin, and glucagon concentrations—if found to be affected in the above time course study—were determined and compared for the late-PN and early-PN groups. Third (Fig. 1), a multivariate mediation analysis [28–30] was then performed on the total study cohort to assess whether any impact of late-PN as compared with early-PN on 3HB could explain its beneficial effects on outcome, adjusted for demographics, baseline risk factors, and type and severity of illness. If so, it was further assessed whether such a mediation role for 3HB was direct or indirect via an effect of late-PN on key regulators of ketogenesis, again adjusted for demographics, baseline risk factors, and type and severity of illness.

**Table 1:** Baseline characteristics of matched patients in the time course study

Baseline characteristics	Early-PN (*n* = 47)	Late-PN (*n* = 49)	*P* value
Age (years)—median [IQR]^2^	4.3 [0.2–8.4]	3.5 [0.4–11.6]	0.4
Age < 1 year—no. (%)	16 (34.0)	18 (36.7)	0.8
Male sex—no. (%)	28 (59.5)	34 (69.3)	0.3
Weight (kg)—median [IQR]^2^	15.0 [4.7–24.0]	14.0 [5.6–34.0]	0.5
STRONGkids risk level—no. (%)^1^			1
Medium (1–3)	44 (93.6)	46 (93.8)	
High (4, 5)	3 (6.3)	3 (6.1)	
PIM2 calc. risk of death (%)—median [IQR]^2^	0.20 [0.08–0.37]	0.16 [0.06–0.33]	0.6
Emergency admission—no. (%)	16 (34.0)	15 (30.6)	0.8
Center—no. (%)			
Leuven	25 (53.1)	26 (53.0)	
Rotterdam	22 (46.8)	23 (46.9)	
Edmonton	0 (0)	0 (0)	
Diagnostic group—no. (%)			
*Surgical*			
Abdominal	0 (0)	2 (4.08)	
Burns	0 (0)	0 (0)	
Cardiac	16 (34.0)	18 (36.7)	
Neurosurgery-traumatic brain injury	8 (17.0)	7 (14.2)	
Thoracic	0 (0)	0 (0)	
Transplantation	4 (8.5)	2 (4.1)	
Orthopedic surgery-trauma	3 (6.3)	0 (0)	
Others	1 (2.1)	0 (0)	
*Medical*			
Cardiac	2 (4.2)	5 (10.2)	
Gastrointestinal-hepatic	0 (0)	0 (0)	
Oncologic-hematologic	0 (0)	1 (2.0)	
Neurologic	1 (2.1)	3 (6.1)	
Respiratory	5 (10.6)	7 (14.2)	
Others	7 (14.8)	4 (8.1)	
Condition on admission—no. (%)			
Need for hemodynamic assist device	1 (0.02)	1 (0.02)	0.2
Presence of infection	26 (55.3)	24 (48.9)	0.5

^1^Scores on the Screening Tool for Risk on Nutritional Status and Growth (STRONGkids) range from 0 to 5, with a score of 0 indicating a low risk of malnutrition, a score of 1 to 3 indicating medium risk, and a score of 4 to 5 indicating high risk. ^2^Pediatric index of mortality 2 (PIM2) is a severity scoring system for predicting outcome of patients admitted to pediatric intensive care units

### Plasma analyses

Plasma insulin and plasma glucagon concentrations were measured with commercial ELISAs (Invitrogen Ltd., Waltham, MA, USA, and Mercodia, Uppsala, Sweden), and blood glucose concentrations with the use of the ABL Radiometer. Plasma 3HB was quantified with a laboratory assay based upon the oxidation of 3HB to acetoacetate by the enzyme 3-hydroxybutyrate dehydrogenase and the concomitant reduction of cofactor NAD+ to NADH [31]. Plasma samples were added to a reaction mixture containing EDTA (2 mM), sodium oxamate (13 mM), NAD (3 mM), and 3-hydroxybutyrate dehydrogenase (1.5 U/ml, 3HBDB-RO Sigma-Aldrich) in Bis-Tris-Propane Buffer (50 mM, pH 9.5). The change in NADH fluorescence, compared to sample with reaction mixture without dehydrogenase, measured in a black 96-well plate with transparent bottom after 15 min at 30 °C, is directly related to the 3HB, with a detection limit of 0.04 mmol/l (excitation 340 nm, emission 445 nm, Tecan Infinite 200, Tecan Ltd., Männedorf, Switzerland).

### Statistical analyses

Data are presented as frequencies and percentages or medians with interquartile range. Fisher’s exact test and Kruskal-Wallis test were used to analyze univariable differences between patient groups, as appropriate. Multivariable Cox proportional hazard analysis was used to assess (first) whether randomization to late-PN or early-PN independently associated with the time to live weaning from mechanical ventilatory support and the time to live discharge from the PICU, and (second) whether 3HB concentrations on the average day of maximal effect (or last day in PICU for patients with a shorter PICU stay) independently associated with these outcomes when added to this model. Likewise, multivariable logistic regression analysis was used to study whether randomization and 3HB concentrations independently associated with the incidence of a new infection in the PICU. Multivariable Cox proportional hazard and logistic regression analyses were adjusted for baseline risk factors (age, weight, gender, emergency versus elective admission, diagnostic group, PIM2 score, STRONGkids category, need of hemodynamic assist device on admission, presence of infection on admission), with censoring performed at 90 days or death. In those cases where adding the plasma 3HB concentration to the multivariable models showed that they were independently associated with the outcome of interest, hereby replacing the effect of the randomization to late-PN versus early-PN on the outcome, the 3HB effect was considered a statistical mediator of the late-PN outcome effect. To assess whether any potential mediator role for 3HB was direct or indirect via an effect of late-PN on key regulators of ketogenesis (plasma insulin, glucagon, and/or blood glucose concentrations) [18], sensitivity analyses were performed in which models were further adjusted for those regulators that were found to be affected by late-PN. In addition, multivariable logistic regression analysis was used to investigate the independent association between plasma 3HB concentration and PICU mortality. All analyses were performed with the use of JMP software, version pro 14 (SAS Institute, NC, USA). Statistical significance was set at a *P* value of 0.0083 for the time course analysis (Bonferroni correction for multiple testing) and at 0.05 for other comparisons.

## Results

### Effect of late-PN versus early-PN on daily plasma concentrations of 3HB and identification of the time point of the maximal effect, if any

The matched cohort of patients with plasma samples available for each of the first 5 PICU days comprised 47 early-PN and 49 late-PN patients (Table 1, Fig. 1). Due to practical reasons, there was a slight time delay between initiation of the randomized intervention at PICU admission and the moment of drawing the admission sample, ranging from 0 to 7 h (median [IQR] 36 [18–74] min).

**Fig. 2:**
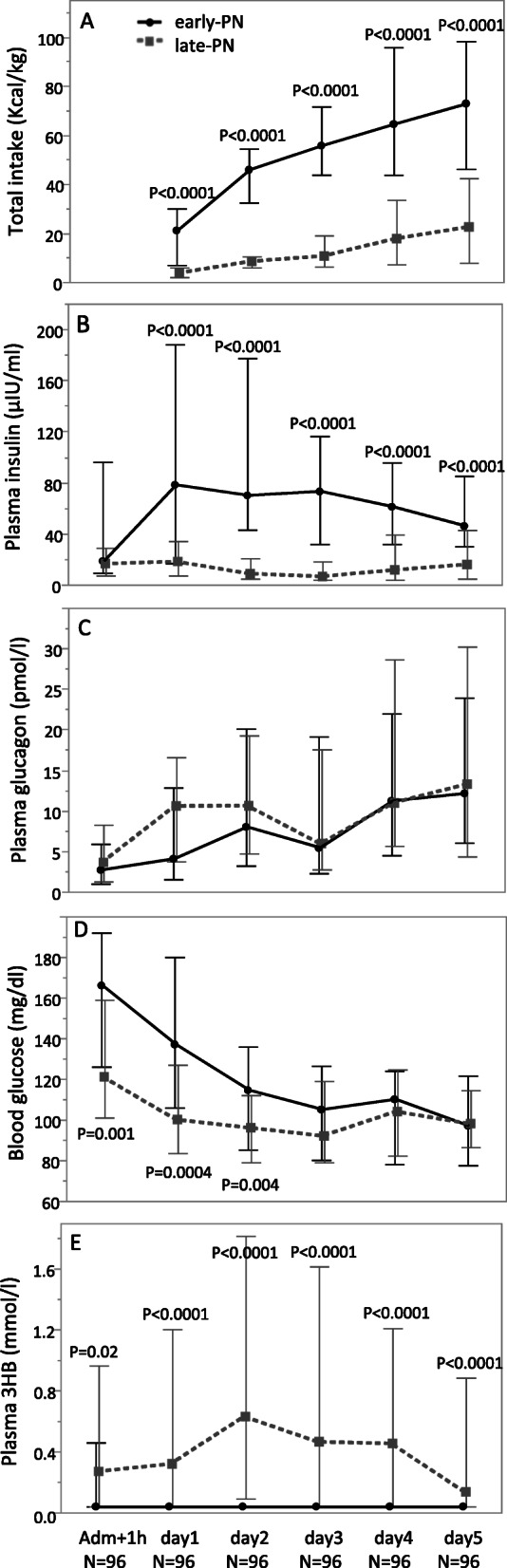
Total caloric intake, plasma insulin, plasma glucagon, blood glucose, and plasma 3HB concentrations during the first 5 days in PICU in a matched cohort of early-PN and late-PN patients. Data are shown as median and IQR

Compliant with the study protocol, the daily total caloric intake was lower in late-PN patients than in early-PN patients throughout the 5 first days in PICU (all *P* < 0.0001) (Fig. 2a). Similar as in the total study population, late-PN patients developed an important macronutrient deficit over the first week in the PICU as compared to early-PN patients. From day 1 to day 5, plasma insulin concentrations were lower in late-PN patients than in early-PN patients (all *P* < 0.0001) (Fig. 2b). Plasma glucagon concentrations were not different (Fig. 2c). From admission until day 2, blood glucose concentrations were lower in the late-PN group (all *P* ≤ 0.004) (Fig. 1d). Throughout the 5 days, plasma 3HB concentrations were higher in late-PN patients than in early-PN patients (*P* = 0.02 for admission and *P* < 0.0001 for days 1 to 5), with the largest difference observed for day 2 (Fig. 2e).

### Effect of late-PN versus early-PN on plasma 3HB concentration on the “maximal effect day” in the total study cohort

Given that day 2 in PICU was identified as the day upon which the difference in median 3HB between late-PN patients and early-PN patients was the largest, plasma 3HB was quantified for all study patients with an available plasma sample on day 2 (*n* = 822). For patients with a PICU stay of less than 2 days, the plasma samples of day 1 were used (*n* = 320) as surrogate.

**Fig. 3:**
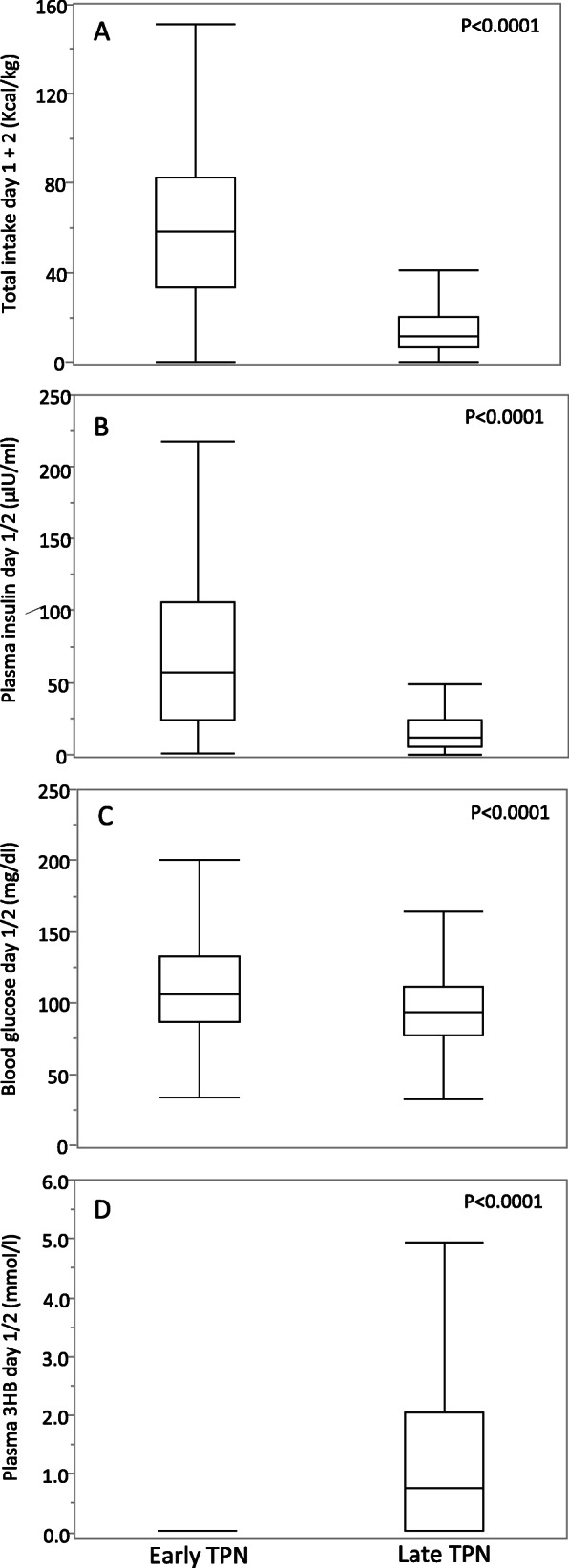
Total caloric intake, plasma insulin, blood glucose, and plasma 3HB concentrations on day 1/2 in the total cohort of early-PN and late-PN patients. Plasma concentrations of 3-HB and insulin were determined on day 2 in PICU or on day 1 for patients with a shorter PICU stay. Boxes show median and IQR, whiskers 10th and 90th percentile

For these 1142 patients, demographics, admission diagnosis, and baseline severity of illness were comparable between the 580 late-PN patients and 562 early-PN patients (Table 2). Cumulative caloric intake until blood sampling was lower in late-PN patients than in early-PN patients (*P* < 0.0001) (Fig. 3a). As in the time course study, plasma insulin and blood glucose concentrations were also lower in late-PN than in early-PN patients (both *P* < 0.0001) (Fig. 3b, c). Given that the time course study did not show an effect of late-PN on plasma glucagon, this analyte was not measured for the entire study cohort. Plasma 3HB concentrations were significantly higher in late-PN than in early-PN patients ((median [IQR]) 0.04 [0.04–0.04] mmol/L versus 0.75[0.04–2.03] mmol/L; *P* < 0.0001) (Fig. 3d).

**Table 2:** Baseline characteristics of patients in the total study cohort

Baseline characteristics	Early-PN (*n* = 580)	Late-PN (*n* = 562)	*P* value
Age (years)—median [IQR]^2^	1.8 [0.3–6.7]	1.9 [0.3–8.4]	0.4
Age < 1 year—no. (%)	238 (41.0)	228 (40.5)	0.9
Male gender—no. (%)	323 (55.6)	324 (57.6)	0.5
Weight (kg)—median [IQR]^2^	11.1 [5.3–21.0]	12.0 [5.1–25.0]	0.3
STRONGkids risk level—no. (%)^1^			0.8
Medium (1–3)	530 (91.3)	511 (90.9)	
High (4, 5)	50 (8.6)	51 (9.0)	
PIM2 calc. risk of death (%)—median [IQR]^2^	0.06 [0.02–0.21]	0.06 [0.02–0.017]	0.5
Emergency admission—no. (%)	272 (46.9)	271 (48.2)	0.6
Center—no. (%)			
Leuven	371 (63.9)	372 (66.1)	
Rotterdam	173 (29.8)	161 (28.6)	
Edmonton	36 (6.2)	29 (5.1)	
Diagnostic group—no. (%)			
*Surgical*			
Abdominal	25 (4.3)	27 (4.8)	
Burns	3 (0.5)	5 (0.8)	
Cardiac	266 (45.8)	255 (45.3)	
Neurosurgery-traumatic brain injury	59 (10.1)	46 (8.1)	
Thoracic	24 (4.1)	20 (3.5)	
Transplantation	7 (1.2)	15 (2.6)	
Orthopedic surgery-trauma	27 (4.6)	26 (4.6)	
Others	11 (1.9)	19 (3.3)	
*Medical*			
Cardiac	22 (3.7)	22 (3.9)	
Gastrointestinal-hepatic	2 (0.3)	3 (0.5)	
Oncologic-hematologic	4 (0.6)	7 (1.2)	
Neurologic	41 (7.0)	36 (6.4)	
Respiratory	54 (9.3)	52 (9.2)	
Others	35 (6.0)	29 (5.1)	
Condition on admission—no. (%)			
Need for hemodynamic assist device	17 (0.0)	23 (0.0)	0.2
Presence of infection	212 (36.5)	193 (34.3)	0.4

^1^Scores on the Screening Tool for Risk on Nutritional Status and Growth (STRONGkids) range from 0 to 5, with a score of 0 indicating a low risk of malnutrition, a score of 1 to 3 indicating medium risk, and a score of 4 to 5 indicating high risk. ^2^Pediatric index of mortality 2 (PIM2) is a severity scoring system for predicting outcome of patients admitted to pediatric intensive care units

### Multivariable outcome analysis to assess whether the impact of late-PN versus early-PN on 3HB could explain its beneficial effects on outcome

In the total study cohort (*n* = 1142), and adjusted for baseline risk factors, randomization to late-PN as compared with early-PN was independently associated with a higher likelihood of an earlier live weaning from mechanical ventilation and earlier live discharge from the PICU and with less newly acquired infections in the PICU (Table 3, panel 1). Adding the plasma 3HB concentrations measured on day 2 (or day 1 for shorter stayers) to the multivariable models revealed that a higher plasma 3HB concentration was independently associated with a higher likelihood of earlier live weaning from mechanical ventilatory support (*P* = 0.0002) and of earlier live PICU discharge (*P* = 0.004), hereby replacing the effect of randomization to late-PN versus early-PN (Table 3, panel 2), thus suggesting a mediator role for the 3HB effect of late-PN on these two outcomes. Further adjustment for the affected key regulators of ketogenesis (plasma insulin concentration, blood glucose concentration) did not alter these results (Table 3, panel 3). The effect of late-PN versus early-PN on plasma 3HB did not explain its impact on infections (Table 3, panels 2 and 3).

**Table 3:** A 3-step approach to investigate a potential mediation role of the effect on 3HB for the outcome benefits of late-PN

	Likelihood of live weaning from mechanical ventilatory support		Likelihood of live discharge from the PICU		Acquisition of a new infection in the PICU	
**A. Impact of randomization (*n*** **= 1142**)	Risk ratio (95% CI)	*P* value	Risk ratio (95% CI)	*P* value	Odds ratio (95% CI)	*P* value
**Randomization to late-PN (*n*** **= 562) vs. early-PN (*n*** **= 580)**	**1.14 (1.01–1.29)**	**0.02**	**1.19 (1.06–1.35)**	**0.003**	**0.49 (0.34–0.70)**	**< 0.0001**
Demographics						
Age per year added	1.02 (0.98–1.05)	0.18	1.02 (0.99–1.06)	0.11	1.02 (0.93–1.13)	0.59
Weight per kg added	0.99 (0.98–1.00)	0.65	0.99 (0.98–1.00)	0.34	0.99 (0.96–1.02)	0.72
Male gender	0.98 (0.85–1.11)	0.85	0.94 (0.83–1.07)	0.39	1.11 (0.78–1.58)	0.52
Characteristics of type and severity of illness						
Emergency vs. planned admission	0.74 (0.61–0.90)	0.003	0.77 (0.63–0.93)	0.008	1.74 (1.03–2.92)	0.03
Diagnostic group		0.22		0.40		0.06
PIM2 score per point added	0.70 (0.66–0.74)	< 0.0001	0.72 (0.68–0.76)	< 0.0001	1.41 (1.25–1.60)	< 0.0001
High vs. medium risk of malnutrition (STRONGkids risk level 4–5 vs. 1–3)	0.86 (0.68–1.06)	0.17	0.83 (0.67–1.03)	0.1	1.15 (0.65–2.02)	0.62
Presence of infection on admission	0.72 (0.61–0.84)	< 0.0001	0.71 (0.61–0.84)	< 0.0001	0.52 (0.32–0.83)	0.005
Need for hemodynamic assist device on admission	0.51 (0.34–0.76)	0.001	0.48 (0.32–0.73)	0.0005	1.71 (0.80–3.64)	0.16
**B. Mediation role for plasma 3HB (*n*** **= 1142)**	Risk ratio (95% CI)	*P* value	Risk ratio (95% CI)	*P* value	Odds ratio (95% CI)	*P* value
**Plasma 3HB on day 2 (per mmol/l)**^**1**^	**1.12 (1.05–1.20)**	**0.0002**	**1.10 (1.03–1.17)**	**0.004**	**0.85 (0.68–1.06)**	**0.14**
**Randomization to late-PN (*n*** **= 562) vs. early-PN (*n*** **= 580)**	**1.01 (0.88–1.16)**	**0.79**	**1.08 (0.94–1.24)**	**0.26**	**0.56 (0.38–0.84)**	**0.004**
Demographics						
Age per year added	1.02 (0.99–1.06)	0.11	1.03 (0.99–1.06)	0.06	1.02 (0.92–1.12)	0.64
Weight per kg added	0.99 (0.98–1.00)	0.55	0.99 (0.98–1.00)	0.28	0.99 (0.96–1.02)	0.72
Male gender	0.99 (0.88–1.12)	0.95	0.95 (0.84–1.07)	0.44	1.11 (0.78–1.57)	0.55
Characteristics of type and severity of illness						
Emergency vs. planned admission	0.77 (0.63–0.94)	0.01	0.79 (0.65–0.96)	0.02	1.65 (0.98–2.79)	0.05
Diagnostic group		0.27		0.32		0.07
PIM2 score per point added	0.70 (0.66–0.74)	< 0.0001	0.72 (0.68–0.76)	< 0.0001	1.41 (1.24–1.60)	< 0.0001
High vs. medium risk of malnutrition	0.85 (0.68–1.05)	0.14	0.83 (0.67–1.03)	0.09	1.16 (0.66–2.05)	0.59
Presence of infection on admission	0.72 (0.61–0.85)	< 0.0001	0.72 (0.61–0.84)	< 0.0001	0.51 (0.32–0.82)	0.004
Need for hemodynamic assist device on admission	0.50 (0.33–0.76)	0.001	0.48 (0.32–0.73)	0.0001	1.66 (0.78–3.55)	0.19
**C. Sensitivity analysis (direct/indirect 3HB effect) (*n*** **= 1142)**	Risk ratio (95% CI)	*P* value	Risk ratio (95% CI)	*P* value	Odds ratio (95% CI)	*P* value
**Plasma 3HB on day 2 (per mmol/l)**^**1**^	**1.12 (1.05–1.19)**	**0.0005**	**1.09 (1.02–1.17)**	**0.007**	**0.80 (0.63–1.02)**	**0.06**
**Randomization to late-PN (*n*** **= 562) vs. early-PN (*n*** **= 580)**	**0.97 (0.83–1.12)**	**0.70**	**1.04 (0.89–1.21)**	**0.60**	**0.62 (0.40–0.94)**	**0.02**
Demographics						
Age per year added	1.03 (0.99–1.06)	0.08	1.03 (0.99–1.06)	0.06	1.03 (0.93–1.14)	0.50
Weight per kg added	0.99 (0.98–1.00)	0.67	0.99 (0.98–1.00)	0.36	0.99 (0.96–1.02)	0.54
Male gender	0.97 (0.86–1.11)	0.74	0.94 (0.83–1.06)	0.34	1.19 (0.83–1.72)	0.33
Characteristics of type and severity of illness						
Emergency vs. planned admission	0.72 (0.59–0.89)	0.002	0.76 (0.62–0.93)	0.01	1.62 (0.94–2.79)	0.07
Diagnostic group		0.28		0.42		0.03
PIM2 score per point added	0.69 (0.65–0.73)	< 0.0001	0.72 (0.68–0.75)	< 0.0001	1.45 (1.27–1.65)	< 0.0001
High vs. medium risk of malnutrition	0.84 (0.67–1.04)	0.12	0.82 (0.65–1.02)	0.07	1.15 (0.64–2.06)	0.63
Presence of infection on admission	0.72 (0.61–0.85)	0.0001	0.72 (0.62–0.85)	0.0001	0.49 (0.30–0.81)	0.004
Need for hemodynamic assist device on admission	0.51 (0.34–0.78)	0.002	0.48 (0.32–0.74)	0.0008	1.44 (0.66–3.13)	0.36
**Late-PN-affected regulators of ketogenesis**						
**Plasma insulin at time of 3HB assessment (per μIU/l)**	**1.00 (0.99–1.00)**	**0.93**	**0.99 (0.99–1.00)**	**0.52**	**0.80 (0.63–1.02)**	**0.76**
**Blood glucose at time of 3HB assessment (per mg/dl)**	**0.99 (0.99–0.99)**	**0.02**	**0.99 (0.99–1.00)**	**0.46**	**0.99 (0.99–1.00)**	**0.49**

^1^Plasma 3HB was quantified for all study patients with an available plasma sample on day 2 (*n* = 822) or day 1 (*n* = 320) for patients with a shorter PICU stay

Plasma 3HB concentrations were not independently associated with PICU mortality (Additional Table 1).

## Discussion

This secondary analysis of the PEPaNIC RCT revealed that late-PN, as compared with early-PN, associated with increased plasma 3HB concentrations from the first hours in PICU onward, sustained for at least 5 days, and with a maximal rise observed on PICU day 2. Also, levels of the key regulators of ketogenesis, blood glucose and insulin, were lowered whereas glucagonemia was unaffected. Statistical mediation analyses suggested that the rise in plasma 3HB concentrations with late-PN, as compared with early-PN, explained its accelerating impact on live weaning from mechanical ventilatory support and on live discharge from the PICU, but not its preventive effect on newly acquired infections. This role of increased 3HB as a potential mediator of 2 major outcome benefits of late-PN was a direct one, as it was independent of the observed changes in the key regulators of ketogenesis.

With late-PN, and thus with accepting an important macronutrient deficit during the first week in the PICU, plasma 3HB concentrations were found to rise up to the millimolar range. This high level of plasma 3HB in response to late-PN was striking given that earlier studies had suggested impaired ketogenesis during critical illness in adults [22–25]. Hyperinsulinemia and hyperglycemia are powerful suppressors of ketogenesis and hallmarks of critical illness [20, 21]. Glucagon, cortisol, and catecholamines are hormones known to enhance ketogenesis in healthy subjects, and levels of these stress hormones are elevated during critical illness [18, 32]. However, as shown previously for cortisol [33], and now also for glucagon, not using early-PN did not affect the plasma levels of these ketogenic hormones in critically ill children. Also, the use of corticosteroids and of catecholamines was unaffected by late-PN, as shown previously [5, 33]. In contrast, not using early-PN lowered blood glucose and plasma insulin concentrations in the current study, in line with the earlier finding of a lowered insulin requirement to prevent hyperglycemia [4, 5]. Together, this suggests that late-PN, as compared with early-PN, increased plasma 3HB concentrations throughout the first 5 days in the PICU by reducing suppressors of ketogenesis rather than by enhancing ketogenesis activators.

With late-PN, the highest rise in plasma 3HB concentrations was observed on day 2 in the PICU with concentrations declining somewhat thereafter, though staying elevated throughout the first 5 days in the PICU. This decline corresponds with the increase in the amount of enteral nutrition from day 2 onwards, although that amount was still small. It is well known that ketogenesis is very sensitive to suppression by macronutrient intake [9]. Also, given that the blood glucose levels were no longer different after PICU day 2, and hyperglycemia is a known suppressor of ketogenesis [22], the blood glucose effect may have contributed to the peak rise in plasma 3HB on that day. Remarkably, although children were not fully fasted during these first PICU days, receiving minimal doses of IV glucose and receiving blood glucose control with insulin infusion, the plasma ketone concentrations in the late-PN group rose quite high, up to the millimolar range. This may have to do with the young age of the critically ill patients of this study, as the ketogenic fasting response in children is known to be more pronounced than in adults [9, 22–25].

The statistical mediation analyses suggested that the rise in plasma 3HB concentrations with late-PN, as compared with early-PN, explained its accelerating impact on live weaning from mechanical ventilatory support and on live discharge from the PICU, but not its preventive effect on newly acquired infections. The mechanism by which 3HB may enhance recovery from critical illness cannot be identified in the current study. One possibility is that 3HB, during critical illness, similarly as during prolonged fasting in healthy subjects, serves as a vital or super fuel for the brain, heart, and skeletal muscle [9]. In healthy athletes, ingestion of 3HB esters has shown to increase endurance [15]. Also, in a mouse model of sepsis, a ketogenic PN formula and supplementation of PN with 3HB have shown to protect against the development of muscle weakness [17]. Alternatively, 3HB may act through enhanced autophagy-driven cellular housekeeping [10], a pathway that was previously shown to be activated by late-PN in critically ill adults [13]. Another possibility is a 3HB-driven activation of muscle regeneration, as was shown earlier in a mouse model of sepsis [11]. Finally, although 3HB may also have anti-inflammatory effects [14], such a mechanism appears less likely, given that late-PN has shown to increase plasma C-reactive protein concentrations, considered to be a marker of inflammation [4, 5]. The finding that the rise in 3HB with late-PN did not explain its preventive impact on new infections also suggests that an immune modulating effect of 3HB does not seem to be involved.

Adjustment for key suppressors of ketogenesis, blood glucose and plasma insulin [18, 20, 22], that were lowered by late-PN, did not affect the statistical mediating role of the rise in plasma 3HB concentrations with late-PN on outcome. This suggests that the effect of the rise in 3HB on accelerated recovery was a direct one, rather than that it merely mirrored the impact of lowered insulin or blood glucose. This was further supported by the finding that plasma insulin concentrations were not independently associated with the late-PN-affected outcomes in the multivariable models. Interestingly, a lower blood glucose concentration was independently associated with a higher likelihood of earlier live weaning from mechanical ventilator support. Targeting normal fasting levels of blood glucose with insulin in adults has previously shown to protect against critical illness-induced polyneuropathy/myopathy, an important cause of limb and respiratory muscle weakness, and a complication that prolongs the need for mechanical ventilation [34, 35]. However, as compared with the large impact on blood glucose in those studies, the difference in blood glucose between late-PN and early-PN that was observed here was small. Altogether, our data open perspectives for studies investigating the impact of exogenous ketone supplementation or ketogenic diets on outcome of critically ill patients. Possibly, such strategies could allow effective and safe initiation of artificial feeding while avoiding prolonged fasting intervals, a hypothesis that requires further study. Evidently, future studies should also investigate potential side effects of such feeding strategies, such as gastrointestinal tolerance and, for ketogenic diets, metabolic acidosis.

One strength of this study is the design as a secondary analysis of a large RCT, with prospectively planned collection of plasma samples on a daily basis. This allowed to perform repeated measurements and to identify the time point of the maximal 3HB effect. This study has also limitations. First, not all patients of the original cohort had a sample available for the day of the maximal 3HB effect. However, samples were available for 1142 of the 1440 patients and the late-PN and early-PN groups were still comparable for demographics, admission diagnosis, and baseline severity of illness. Also, the outcome benefits of late-PN versus early-PN were present in the cohort of 1142 patients and comparable to those in the 1440 patient cohort. A second limitation is that the mediation analyses were performed with plasma 3HB concentrations quantified on a single time point, namely the day of the maximal 3HB effect. It thus remains unclear whether the observed positive effect of plasma 3HB concentrations on outcome is related to its peak effect or to the duration of enhanced ketogenesis during critical illness. Third, in the sensitivity mediation analyses, in which it was investigated whether the impact of late-PN versus early-PN on plasma insulin and blood glucose played a role, it cannot be excluded that other, yet unknown, factors were involved. Finally, this study was performed in a pediatric ICU population. Whether these findings can be extrapolated to adult ICU patients requires further investigation.

## Conclusion

Withholding PN during the first week in the PICU, whereby suppressors of ketogenesis were reduced, rapidly increased plasma 3HB concentrations up to the millimolar range, an effect that was sustained for at least 5 days. This 3HB effect of withholding PN was found to be a direct statistical mediator of an important part of its beneficial impact on recovery. These findings open perspectives for the further investigation of ketone supplementation and of ketogenic diets to improve outcome of critically ill patients.

## Supplementary information

**Additional file 1: Table 1.** Association of plasma 3HB concentration with PICU mortality.

## Data Availability

Data sharing is offered under the format of collaborative projects. Proposals can be directed to the corresponding author.
