# Supplementary material for: Effect of withholding early parenteral nutrition in PICU on ketogenesis as potential mediator of its outcome benefit
Source: Crit Care. 2020 Aug 31;24:536. doi: 10.1186/s13054-020-03256-z (PMC7456767; doi:10.1186/s13054-020-03256-z)
Supplement: Supplementary file 1 — Additional file 1: Table 1. Association of plasma 3HB concentration with PICU mortality. [file 13054_2020_3256_MOESM1_ESM.docx]

**Additional table 1.** Association of plasma 3HB concentration with PICU mortality

| 1. Association randomization and PICU mortality | Risk ratio (95% CI) | P-value |
| --- | --- | --- |
| Randomization to late PN vs. early PN | **0.68 (0.33-1.38)** | **0.28** |
| Demographics |  |  |
| Age per year added | 0.94 (0.76-1.15) | 0.55 |
| Weight per kg added | 1.02 (0.96-1.08) | 0.41 |
| Male gender | 0.89 (0.45-1.79) | 0.76 |
| Characteristics of type and severity of illness |  |  |
| Emergency vs. planned admission | 2.50 (0.75-8.32) | 0.13 |
| Diagnostic group |  | 0.71 |
| PIM2 score per point added | 2.35 (1.86-2.97) | <0.0001 |
| High vs. medium risk of malnutrition | 1.07 (0.35-3.30) | 0.89 |
| Presence of infection on admission | 2.11 (0.82-5.44) | 0.11 |
| Need for haemodynamic assist device on admission | 2.32 (0.87-6.15) | 0.09 |
| 2. Association 3HB and PICU mortality | **Risk ratio (95% CI)** | **P-value** |
| Plasma 3HB on day 2 (or day 1 for shorter stayers) (per mmol/l added) | **0.59 (0.32-1.11)** | **0.07** |
| Randomization to late PN vs. early PN | **0.88 (0.41-1.90)** | **0.76** |
| Demographics |  |  |
| Age per year added | 0.93 (0.76-1.14) | 0.50 |
| Weight per kg added | 1.02 (0.96-1.08) | 0.41 |
| Male gender | 0.91 (0.45-1.82) | 0.79 |
| Characteristics of type and severity of illness |  |  |
| Emergency vs. planned admission | 2.16 (0.64-7.25) | 0.20 |
| Diagnostic group |  | 0.70 |
| PIM2 score per point added | 2.31 (1.83-2.92) | <0.0001 |
| High vs. medium risk of malnutrition | 1.10 (0.35-3.46) | 0.86 |
| Presence of infection on admission | 2.11 (0.81-5.50) | 0.12 |
| Need for haemodynamic assist device on admission | 2.14 (0.79-5.76) | 0.13 |
| 3. Sensitivity analysis | **Risk ratio (95% CI)** | **P-value** |
| Plasma 3HB on day 2 (or day 1 for shorter stayers) (per mmol/l added) | **0.66 (0.35-1.22)** | **0.12** |
| Randomization to late PN vs. early PN | **1.01 (0.45-2.28)** | **0.96** |
| Demographics |  |  |
| Age per year added | 0.91 (0.74-1.12) | 0.41 |
| Weight per kg added | 1.03 (0.97-1.09) | 0.30 |
| Male gender | 0.87 (0.42-1.79) | 0.71 |
| Characteristics of type and severity of illness |  |  |
| Emergency vs. planned admission | 2.21 (0.64-7.59) | 0.20 |
| Diagnostic group |  | 0.41 |
| PIM2 score per point added | 2.32 (1.82-2.97) | <0.0001 |
| High vs. medium risk of malnutrition | 1.54 (0.49-4.78) | 0.46 |
| Presence of infection on admission | 2.05 (0.78-5.41) | 0.14 |
| Need for haemodynamic assist device on admission | 2.49 (0.90-6.90) | 0.08 |
| Late PN-affected key regulators of ketogenesis |  |  |
| Plasma insulin at time of 3HB assessment (per µIU/l added) | 1.00 (0.99-1.00) | 0.25 |
| Blood glucose at time of 3HB assessment (per mg/dl added) | 1.00 (0.99-1.01) | 0.74 |
